# Being aware of the painful body: Validation of the German Body Awareness Questionnaire and Body Responsiveness Questionnaire in patients with chronic pain

**DOI:** 10.1371/journal.pone.0193000

**Published:** 2018-02-28

**Authors:** Holger Cramer, Romy Lauche, Jennifer Daubenmier, Wolf Mehling, Arndt Büssing, Felix J. Saha, Gustav Dobos, Stephanie A. Shields

**Affiliations:** 1 Department of Internal and Integrative Medicine, Kliniken Essen-Mitte, Faculty of Medicine, University of Duisburg-Essen, Essen, Germany; 2 Australian Research Centre in Complementary and Integrative Medicine (ARCCIM), University of Technology Sydney, Sydney, Australia; 3 Holistic Health Studies, Department of Health Education, San Francisco State University, San Francisco, CA, United States of America; 4 Osher Center for Integrative Medicine, University of California San Francisco (UCSF), San Francisco, CA, United States of America; 5 Center for Integrative Medicine, Faculty of Medicine, University of Witten/Herdecke, Herdecke, Germany; 6 Department of Psychology, Pennsylvania State University, State College, PA, United States of America; TNO, NETHERLANDS

## Abstract

Body awareness is an attentional focus on and awareness of internal body sensations. This study aimed to validate German versions of the Body Awareness Questionnaire (BAQ) and the Body Responsiveness Questionnaire (BRQ) in chronic pain patients and to assess their associations with pain-related variables and to assess their responsiveness to intervention. The instruments were translated to German and administered to 512 chronic pain patients (50.3±11.4 years, 91.6% female) to assess their factor structure and reliability. Cronbach’s α for the BAQ total score was 0.86. Factor analysis of the BRQ revealed the two factors *Importance of Interoceptive Awareness* (Cronbach’s α = 0.75) and *Perceived Connection* (Cronbach’s α = 0.75) and the single-item *Suppression of Bodily Sensations*. The BAQ was independently associated with lower mindfulness, self-esteem, stress, and depression; *Importance of Interoceptive Awareness* with mindfulness, self-acceptance, self-esteem, and physical contact; *Perceived Connection* with self-acceptance, vitality, and lower sensory pain; *Suppression of Bodily Sensations* with lower self-esteem, physical contact, and higher depressive symptoms. After a 10-week multimodal mind-body program (n = 202), the BAQ and *Importance of Interoceptive Awareness* increased and pain intensity and *Suppression of Bodily Sensation* decreased. In conclusion, body awareness and body responsiveness are associated with pain-related variables in patients with chronic pain. Mind-body interventions may positively influence both pain and body awareness, hinting at a potential mechanism of action of these interventions to be tested in further research.

## Introduction

Body awareness is an attentional focus on and awareness of internal body sensations [1]. In a more elaborate conception, body awareness is a multi-dimensional construct that refers to the subjective, phenomenological aspect of proprioception and interoception that enters conscious awareness, which is modifiable by mental processes including attention, interpretation, appraisal, beliefs, memories, conditioning, attitudes and affect [1].When taken as an intense focus on somatic symptoms, body awareness has long been associated with rumination, catastrophizing, and somatization [2], attentional strategies that increase the risk of chronic pain [3]. Distraction from pain, rather than awareness of it, has been shown to be beneficial for coping with pain [4]. On the other hand, focusing on the sensory dimension of pain rather than on the emotional dimension has been shown to yield favorable outcomes for patients with chronic pain conditions [5,6]. Similarly, ‘interoceptive exposure’ has been suggested recently as an innovative approach to chronic pain conditions and shown preliminary benefits for pain-related distress [7]. In this view, body awareness can take the form of a mindful, non-evaluative awareness of subtle body cues or sensations of discomfort and pain without overreacting to them [1]. While the concept of body awareness overlaps with that of mindfulness, body awareness has been defined as either a prerequisite for mindfulness [8], or a more specific construct that shares similarities with mindfulness but is not commonly assessed by standard mindfulness instruments [1]. In empirical research, the two constructs can be differentiated; it has e.g. been shown that associations of exercise with eating behavior are mediated by body awareness but not by mindfulness [9].

While the association of different attentional strategies towards pain has been extensively investigated [10–13], little is known regarding associations of general body awareness and body responsiveness with chronic pain. It has been shown that primary care patients with low back pain with a higher tendency not to worry about sensations of pain or discomfort have a lower tendency to catastrophize and tend to ignore their pain, whereas individuals with training in mind-body approaches tend to catastrophize less while not distracting themselves from sensations of pain or discomfort [14]. Interestingly, the mere awareness of bodily sensations was not associated with pain-related symptoms, cognitions, or coping styles [14]. Related to body awareness but conceptually distinct, body responsiveness is the tendency to integrate body sensations into conscious awareness to guide decision-making and behavior and not suppress or react impulsively to them [15,16]. Greater body responsiveness has been associated with greater body satisfaction and e.g. less disordered eating [15] however its association with chronic pain, that is generally associated with negative bodily cues, has not been investigated.

A number of instruments assessing body awareness have been developed, with the Body Awareness Questionnaire (BAQ) being the most commonly used [1]. On the other hand, only one instrument, the Body Responsiveness Questionnaire (BRQ) explicitly assesses body responsiveness [1]. Neither the BAQ nor the BRQ have been validated in patients with pain.

The first aim of this study was to validate the German versions of the BAQ and the BRQ in patients with chronic pain conditions, i.e. to assess their construct validity, reliability and convergent validity in this patient group. The second aim was to assess associations of pain intensity and pain-related variables with body awareness and body responsiveness. We hypothesized negative associations between the two categories of variables. Finally, the study aimed to assess responsiveness of the two instruments, that is, sensitivity to change from a mind-body intervention in patients with chronic pain. We hypothesized increases in body awareness and body responsiveness following the intervention.

## Materials and methods

### Sample and setting

All patients with an ICD-10 diagnosis of a chronic pain condition (i.e., spinal pain, fibromyalgia, headache, osteoarthritis, arthritis, or other chronic pain conditions) who were referred to inpatient or outpatient treatment at the Department of Internal and Integrative Medicine, Faculty of Medicine, University of Duisburg-Essen, Germany between January 2013 and July 2014 were invited to participate in this study. Written informed consent was obtained and patients completed questionnaires immediately at their admission. Outpatients additionally completed questionnaires after the end of treatment in order to analyze responsiveness (see below). The study was planned and conducted in accordance with the principles of the World Medical Association’s declaration of Helsinki and the German Medical Association's Professional Code of Conduct and was approved by the Ethics Committee of the University of Duisburg- Essen (approval numbers 12-5216-BO and 13-5393-BO) before patient recruitment.

### Measures

#### Body Awareness Questionnaire (BAQ)

The BAQ measures attentiveness to normal, non-emotive internal bodily processes and sensations, specifically sensitivity to bodily cycles and rhythms, small changes in normal functioning, and anticipation of bodily reactions on 18 items scored on a 7-point Likert scale ranging from 1 (not at all true about me) to 7 (very true about me). The original version of the BAQ has four scales: “note responses or changes in body process”; “predict bodily reaction”; “sleep-wake cycle”; and “onset of illness”; but normally a total score is calculated [16]. The original English language instrument was translated into German independently by two German native speakers with intensive English language training and knowledge of the English-speaking culture (HC, RL). Both translators were health professionals and experienced in assessing questionnaire and interview data. The translation aimed at a conceptual equivalent of the respective item rather than a word-for-word translation. Both translations were combined by the translators into a single consensus translation by discussion. The instrument was then back-translated into English by two independent professional translators who had no knowledge of the original instrument and had no health background. Again, a single back-translation was produced by discussion until consensus was reached. Concordance of the back-translated version and the original BAQ was discussed by the translators, the developer of the original instrument (SS) and an English native speaking Professor of German Language (Dr. Lewis Jillings). This discussion resulted in slightly changing item 18 from “Ich bemerke spezifische Körperreaktionen, wenn ich ausgehungert bin.”to “Ich bemerke spezifische Körperreaktionen, wenn ich übermäßig hungrig bin.“, more closely reflecting the original wording „over-hungry“.

#### Body Responsiveness Questionnaire (BRQ)

The BRQ is a 7-item instrument measuring responsiveness to bodily sensations on a 7-point Likert scale ranging from 1 (not at all true about me) to 7 (very true about me) [15]. A factor analysis found a two-factor solution of the BRQ with the factors, *Importance of Interoceptive Awareness*, and *Perceived Disconnection* [1,17]. The first factor assesses the importance of using interoceptive information to regulate behavior and self-awareness and the second factor assesses the degree of disconnection between psychological and physical states.

The original English language instrument was translated and back-translated following the similar procedure as the BAQ. Concordance of the back-translated version and the original BAQ was discussed by the translators, the developer of the original instrument (JD) and a German native speaking expert in developing and validating English language questionnaires (WM). Based on this discussion, item 3 „Mein Verstand und mein Körper wollen oft zwei verschiedene Dinge tun.”was slightly changed to „Mein Geist und mein Körper wollen oft zwei verschiedene Dinge tun.“, more closely reflecting the original wording „mind“; and item 4 „Ich unterdrücke meine körperlichen Gefühle und Empfindungen.”to „Ich unterdrücke meine körperlichen Empfindungen und Wahrnehmungen.“, more closely reflecting the original wording „feelings and sensations“.

#### Dresden Body Image Inventory (Dresdner Körperbildfragebogen; DKB)

The DKB measures body image as body-related self-perceptions and self-attitudes. This concept includes body-related thoughts, beliefs, feelings, and behaviors. The DKB assesses body image on 5 scales: vitality; self-acceptance; sexuality; self-esteem; and physical contact by 35 items scored on a 5-point Likert scale ranging from 1 (not at all) to 5 (totally) [18].

#### Conscious Presence and Self Control (CPSC)

The CPSC is a modified form of the Freiburg Mindfulness Inventory, measuring situational awareness (‘mindfulness’) by 10 items on a 4-point Likert scale ranging from 0 (rarely) to 3 (almost always) [19]. To respond to the items, participants neither require formal mindfulness / meditation training nor specific knowledge of the underlying (philosophical) concepts.

#### Visual analog scale (VAS)

Mean pain intensity during the past 4 weeks was measured on a 100-mm VAS ranging from 0 (no pain at all) to 100 (worst pain imaginable) based on the German Pain Questionnaire [20].

#### Pain Perception Scale (PPS)

The PPS measures the subjective experience of pain on two scales: affective pain and sensory pain with 24 items on a 4-point Likert scale ranging from 1 (does not apply at all) to 4 (totally applies) [21].

#### Pain Disability Index (PDI)

The PDI assesses how much specific aspects of a person’s life are disrupted by chronic pain with 7 items on an 11-point Likert scale from 0 (no disability) to 10 (worst disability) [22].

#### Beck Depression Inventory (BDI)

Depressive symptoms were assessed by the 21-item BDI using 4-point Likert scales from 0 to 3 [23,24].

#### Perceived Stress Scale (PSS)

General perceptions of stress during the last month were assessed using the 10-item German version of the PSS using a 4-point Likert scale from 1 (never) to 4 (very often) [19].

### Statistical analysis

#### Construct validity

To explore each instrument’s structure, an exploratory factor analysis using the maximum likelihood method and promax rotation was performed separately on the 18 items of the BAQ and the 7 items of the BRQ. Factors were extracted if their eigenvalue was >1. Domain scores of any resulting factors, or of a total score, were calculated as the sum of the component item scores.

### Reliability

To assess internal consistency of the BAQ and the BRQ, Cronbach's α, alpha if item deleted, item-scale correlations, and item difficulty were calculated for each factor and the total score. Split-half reliability was assessed as the Spearman-Brown coefficient. Two-way random intra-class correlation (ICC_2,1_) and its 95% confidence interval [2] was used to assess agreement between measures.

#### Convergent validity

As a measure of convergent validity, the strength of relationship of the BAQ and the BRQ with theoretically related instruments for measures of body image and mindfulness was assessed. Pearson's correlation coefficients between the two instruments and the DKB and CPSC were calculated.

### Body awareness and body responsiveness in patients with chronic pain

To assess body awareness and body responsiveness in patients with chronic pain, the instruments’ total scores and/or subscale scores were assessed and differences between setting (inpatient versus outpatient) and gender (men versus women) were tested using independent *t*-tests. Associations of the BAQ and the BRQ with measures of body image and mindfulness (DKB, CPSC), as well as with clinical measures of pain (VAS, PPS, PDI) and mood (BDI, PSS) were assessed by Pearson's correlation coefficients. Additionally, independent predictors of body awareness and body responsiveness were assessed by linear forward stepwise multiple regression analyses with linear outcome and linear or dichotomous regressors. Only variables that were significantly correlated with the respective instrument or subscale in univariate analysis were included in the initial regression model.

#### Responsiveness

Sensitivity to change was calculated in the outpatient subsample. After the first assessment, these patients participated in a 10-week mind-body intervention [25]. The 60-hour program was delivered in a semi-residential clinic for 6 hours on one day each week over 10 weeks and consisted of stress management training, moderate exercise, Mediterranean diet, and cognitive behavioral techniques with a focus on self-care strategies. The intervention is based on the mind–body program of the Benson–Henry Mind/Body Medical Institute, Harvard Medical School [26] and the Mindfulness-Based Stress Reduction Program of the University of Massachusetts [27,28]. The intervention has been shown to decrease pain intensity in patients with chronic pain [29]; in addition to teaching mindfulness, it incorporates a number of interventions that have been shown to improve body awareness, such as yoga and qigong/Tai chi [30–31]. These interventions were practiced about 60 to 120 minutes during each meeting, and patients were encouraged to also practice at home [25]. Values of BAQ and BRQ before and after the mind body program were compared using a paired-sample *t*-test. Differences in baseline characteristics between patients who dropped-out of the intervention and completers where tested using independent-sample *t*-tests or Chi-squared tests as appropriate.

All statistics were performed using the statistical package IBM SPSS Statistics (Version 20.0; IBM Inc., New York, USA). A *p*-value of <0.05 (two-tailed) was considered significant in all analyses.

## Results

### Sociodemographic characteristics

The psychometric sample consisted of 512 patients of which 469 (91.6%) were female. Age ranged from 19 to 75 years with a mean age of 50.3±11.4 years. Mean pain intensity was 45.2 mm±26.1 mm on the VAS; mean pain duration was 12.9±11.9 years (Table 1).

**Table 1:** Sociodemographic and clinical characteristics of the psychometric sample (mean±standard deviation).

Variable	Total (*N* = 512)	Inpatient sample (*n* = 310)	Outpatient sample (*n* = 202)
**Sociodemographic characteristics**			
Age, in years	50.3±11.4	50.7±12.4	49.6±9.7
Gender			
Female, *n* (%)	469 (91.6%)	282 (91.0%)	187 (92.6%)
BMI, in kg/m^2^	26.4±5.6	26.9±5.7	25.7±5.2
Family status, *n* (%)			
Single	87 (17.0%)	57 (18.4%)	30 (14.9%)
With partner/married	338 (66.0%)	193 (62.3%)	145 (71.8%)
Divorced, separated, widowed	81 (15.8%)	54 (17.4%)	27 (13.4%)
Education, *n* (%)			
< High school	279 (54.5%)	171 (55.2%)	108 (53.5%)
At least high school	124 (24.2%)	73 (23.5%)	51 (25.2%)
University	106 (20.7%)	63 (20.3%)	43 (21.3%)
Employment, *n* (%)			
Full-time	170 (33.2%)	95 (30.6%)	75 (37.1%)
Part-time	132 (25.8%)	72 (23.22%)	60 (29.7%)
Unemployed	26 (5.1%)	20 (6.5%)	6 (3.0%)
Home keeper	32 (6.3%)	21 (6.8%)	11 (5.4%)
Retired	96 (18.8%)	68 (21.9%)	28 (13.9%)
Sick leave	48 (9.4%)	27 (8.7%)	21 (10.4%)
Student	3 (0.6%)	2 (0.6%)	1 (0.5%)
Applied for disability pension, *n* (%)	83 (16.2%)	52 (16.8%)	31 (15.3%)
**Clinical characteristics**			
Pain condition, N (%)^a^			
Headache			
Migraine	105 (20.5%)	39 (12.6%)	66 (32.7%)
Tension type headache	33 (6.4%)	11 (3.5%)	22 (10.9%)
Other headache	80 (15.6%)	50 (16.1%)	30 (14.9%)
Rheumatic diseases			
Fibromyalgia	114 (22.3%)	71 (22.9%)	43 (21.3%)
Osteoarthritis	90 (17.6%)	57 (18.4%)	33 (16.3%)
Rheumatoid arthritis	17 (3.3%)	10 (3.2%)	7 (3.5%)
Spinal/shoulder pain			
Low back pain	150 (29.3%)	82 (26.5%)	68 (33.7%)
Neck pain	48 (9.4%)	29 (9.4%)	19 (9.4%)
Shoulder pain	55 (10.7%)	31 (10.0%)	24 (11.9%)
Other pain	265 (51.8%)	175 (56.5%)	90 (44.6%)
Duration of pain, in years	12.9±11.9	11.0±10.7	15.6±13.0
Intensity of pain, 0-100mm VAS	45.2±26.1	48.2±25.2	40.7±26.9

^a^More than one pain condition per patient possible.

### Descriptive scale characteristics, factor structure and reliability

#### BAQ

Mean item values ranged from 3.1 to 5.6 (Table 2). For all items, the whole 1–7 range of the item was scored by > 10 patients. Kaiser-Meyer-Olkin’s measure of sampling adequacy was good with 0.86, indicating that the sample was suitable for factor analysis. Maximum likelihood factor analysis revealed a four-factor structure which would explain 50.7% of the variance (Table 2). Cronbach’s *α* for the individual factors were low and ranged from 0.52 to 0.79. Cronbach’s *α* was 0.84 for the single-factor solution; since this would also increase comparability with the original instrument, a single-factor solution was preferred. For all items, deletion of a specific item would have reduced or not affected Cronbach’s *α* except for the item “Seasonal rhythms”, which deletion would have improved alpha (Table 2). Due to the low corrected item total correlation of this item, it was excluded from the final instrument. Testing for split-half reliability of the remaining items revealed a Spearman-Brown coefficient of 0.79. Intra-class correlation was ICC_2,1_ = 0.86, 95% CI = 0.84 to 0.88.

**Table 2:** Descriptive scale characteristics, factor structure, and reliability of the German version of the Body Awareness Questionnaire (BAQ).

	Item	Mean± standard deviation	Item difficulty	Loading (alpha = 0.84)^a^	Corrected item-total correlation^a^	Alpha if item deleted^a^	Loading factor 1 (alpha = 0.79)	Loading factor 2 (alpha = 0.69)	Loading factor 3 (alpha = 0.52)	Loading factor 4 (alpha = 0.70)
1	Reactions to food types	4.7±1.9	0.67	0.39	0.38	0.84	-	-	0.68	-
2	Predict bruise	4.3±2.1	0.61	0.48	0.44	0.84	0.41	-	-	-
3	Predict muscle soreness	4.7±1.7	0.67	0.56	0.51	0.83	0.50	-	-	-
4	Food and energy level	3.7±1.8	0.53	0.50	0.47	0.84	-	-	0.72	-
5	Predict the flu	3.6±1.9	0.51	0.53	0.50	0.84	-	-	-	0.86
6	Detect fever	4.1±2.1	0.59	0.52	0.47	0.83	-	-	-	0.67
7	Fatigue of hunger versus sleepiness	5.2±1.7	0.74	0.59	0.53	0.84	0.66	-	-	-
8	Predict lack of sleep effects	4.1±1.9	0.59	0.60	0.54	0.83	0.57	-	-	-
9	Activity cycle in day	4.8±1.6	0.69	0.56	0.51	0.83	0.62	-	-	-
10	Seasonal rhythms^b^^,^^c^	4.6±1.9	0.66	0.01	0.02	0.86	-	-	0.11	-
11	Predict energy level	3.7±1.8	0.53	0.56	0.50	0.83		0.70	-	-
12	Predict quality of sleep	3.1±1.7	0.44	0.49	0.42	0.84	-	0.71	-	-
13	Reaction to fatigue	5.6±1.4	0.80	0.52	0.48	0.84	0.54	-	-	-
14	Reaction to weather	4.9±1.8	0.70	0.38	0.37	0.84	-	-	0.43	-
15	Predict sleep needs	3.6±1.9	0.67	0.55	0.49	0.84	-	0.57	-	-
16	Exercise and energy level	4.1±1.6	0.61	0.58	0.53	0.83	0.58	-	-	-
17	Timing of sleep	4.4±1.8	0.67	0.45	0.42	0.84	0.44	-	-	-
18	Reaction to hunger	5.1±1.6	0.53	0.50	0.46	0.84	0.56	-	-	-

^a^One-factor solution.

^b^Reversed coding.

^c^Item excluded from final instrument.

#### BRQ

Mean item values ranged from 3.1 to 4.9 (Table 3). For all items, the whole 1–7 range was scored by > 10 patients. Kaiser-Meyer-Olkin’s measure was moderate with 0.71. The factor analysis revealed a two-factor structure explaining 61.9% of the variance (Table 4). Cronbach’s *α* was 0.75 for factor 1 *Importance of Interoceptive Awareness* but only 0.63 for factor 2 *Perceived Connection* (the factor was slightly renamed from the English original in order to reflect that higher reverse-scored values represent higher body responsiveness). The exclusion of the item “Suppression of bodily sensations” (hereafter treated as a non-reversed single-item factor) from factor 2 however increased *α* to 0.75. For factor 1, Spearman-Brown coefficient was 0.71, intra-class correlation was ICC_2,1_ = 0.75, 95% CI = 0.71 to 0.78. For factor 2, Spearman-Brown coefficient was 0.75, intraclass correlation was ICC_2,1_ = 0.75, 95% CI = 0.70 to 0.79.

**Table 3:** Pearson correlations of the body awareness questionnaire and the body responsiveness questionnaire with measures of body image and mindfulness.

	Body Awareness Questionnaire	Body Responsiveness Questionnaire			Dresden Body Image Inventory					Conscious Presence and Self Control
		Importance of Interoceptive Awareness	Perceived Connection	Suppression of Bodily Sensations	Vitality	Self-accep-tance	Sexuality	Self-esteem	Physical contact	
**Body Awareness Questionnaire**	-	0.45	-0.18	0.03	-0.00	0.14	0.05	0.16	0.07	0.27
**Body Responsiveness Questionnaire**										
Importance of Interoceptive Awareness	0.45	-	0.06	-0.28	0.19	0.34	0.20	0.28	0.18	0.39
Perceived Connection	-0.18	0.06	-	-0.38	0.26	0.30	0.14	0.18	0.09	0.20
Suppression of bodily sensations	0.03	-0.28	-0.38	-	-0.18	-0.28	-0.24	-0.19	-0.24	-0.25

**Table 4:** Descriptive scale characteristics, factor structure, and reliability of the German version of the Body Responsiveness Questionnaire (BRQ).

				Factor 1: Importance of Interoceptive Awareness (alpha = 0.75)				Factor 2: Perceived Connection (alpha = 0.63)		
	Item	Mean± standard deviation	Item difficulty	Factor loading	Alpha if item deleted		Corrected item-total correlation	Factor loading	Alpha if item deleted	Corrected item-total correlation
1	Body lets me know what is good for me	4.9±1.5	0.70	0.44		0.77	0.40	-	-	-
2	Regret results of bodily^a^ desires	3.7±1.8	0.53	-		-	-	0.74	0.54	0.55
3	Mind and body want different things^a^	3.1±1.7	0.44	-		-	-	0.79	0.48	0.60
4	Suppression of bodily sensations^b^	4.0±1.8	0.57	-		-	-	0.50	0.75	0.38
5	Listen to my body	4.0±1.5	0.57	0.61		0.71	0.52	-	-	-
6	Important to know how my body feels	4.5±1.5	0.64	0.79		0.64	0.65	-	-	-
7	Enjoy becoming aware of my body	4.1±1.6	0.59	0.80		0.64	0.64	-	-	-

^a^Reversed coding.

^b^Single-item factor.

### Convergent validity

Results of the correlation analyses to determine the convergent validity of the BAQ and BRQ are shown in Table 3. BAQ was moderately correlated with the BRQ subscale *Importance of Interoceptive Awareness* and weakly negatively correlated with *Perceived Connection*. Further, BAQ was weakly correlated with the DKB subscales *Self-acceptance* and *Self-esteem* as well as with mindfulness. The BRQ subscales and the single item *Suppression of Bodily Sensations* were weakly to moderately correlated with all DKB subscales (except for *Perceived Connection* with *Physical Contact*) and with mindfulness.

### Body awareness and body responsiveness in patients with chronic pain

#### BAQ

The mean BAQ total score was 73.4±16.8 and did not differ between settings (inpatients versus outpatients) or genders (Table 5). Regarding clinical measures, BAQ was weakly correlated with sensory pain on the PPS only (Table 6). In regression analysis (*r*^2^ = 0.15), body awareness was independently associated with setting (*p*<0.001; higher values in outpatients), mindfulness on the CPSC (*p*<0.001), self-esteem on the DKB (*p*<0.001), perceived stress (*p* = 0.004), and depression (*p* = 0.015) (Table 7).

**Table 5:** Total score of the instruments or scales (mean±standard deviation) in the complete sample and differences between inpatients and outpatients; and between men and women.

	Total sample (*n* = 512)	Inpatient sample (*n* = 310)	Outpatient sample (*n* = 202)	*P*	Men (*n* = 42)	Women (*n* = 470)	*P*
**Body Awareness Questionnaire**	73.4±16.8	72.7±16.8	74.4±16.8	0.260	69.0±14.9	73.8±16.9	0.076
**Body Responsiveness Questionnaire**							
Importance of Interoceptive Awareness	17.5±4.6	17.5±4.7	17.5±4.4	0.972	17.2±4.1	17.5±4.7	0.727
Perceived Connection	6.8±3.2	7.0±3.2	6.4±3.2	0.030	8.1±3.1	6.7±3.2	0.006
Suppression of Bodily Sensations	3.9±1.8	4.1±1.8	3.8±1.8	0.106	4.0±1.5	3.9±1.8	0.992

**Table 6:** Pearson correlations of the Body Awareness Questionnaire and the Body Responsiveness Questionnaire with clinical measures of pain and mood.

	Pain Intensity	Pain Perception Scale		Pain Duration	Pain Disability Index	Beck Depression Inventory	Perceived Stress Scale
		Affective pain	Sensory pain				
**Body Awareness Questionnaire**	0.01	0.05	0.13	0.08	0.07	0.02	0.03
**Body Responsiveness Questionnaire**							
Importance of Interoceptive Awareness	-0.03	-0.04	0.02	0.07	-0.04	-0.20	-0.19
Perceived Connection	-0.04	-0.14	-0.12	-0.04	-0.11	-0.24	-0.23
Suppression of Bodily Sensations	-0.07	0.08	0.12	0.09	0.10	0.33	0.27

**Table 7:** Independent predictors of body awareness on the Body Awareness Questionnaire (BAQ) and body responsiveness on the Body Responsiveness Questionnaire (BRQ).

Independent variable	Predictor variable	B ±SE	β	p	Adjusted R^2^
**Body Awareness** **Questionnaire**	Constant	27.4±7.8		<0.001	**0.15**
	Mindfulness	0.3±0.1	0.3	<0.001	
	Perceived stress	0.6±0.2	0.2	0.004	
	Self-esteem	6.2±1.4	0.3	<0.001	
	Setting	7.6±1.9	0.2	<0.001	
	Depression	0.3±0.1	0.1	0.015	
**Body Responsiveness Questionnaire**					
Importance of Interoceptive Awareness	Constant	6.5±1.3		<0.001	**0.21**
	Mindfulness	0.1±0.0	0.3	<0.001	
	Self-esteem	0.9±0.3	0.1	0.006	
	Self-acceptance	0.8±0.4	0.1	0.036	
	Physical contact	0.6±0.3	0.1	0.041	
Perceived Connection	Constant	2.2±1.1		0.034	**0.16**
	Self-acceptance	1.1±0.2	0.2	<0.001	
	Vitality	0.8±0.2	0.2	0.001	
	Gender	1.3±0.5	0.1	0.007	
	Sensory pain	-0.1±0.0	-0.1	0.015	
	Setting	0.7±0.3	0.1	0.020	
Suppression of Bodily Sensations	Constant	5.8±0.6		<0.001	**0.16**
	Depression	0.1±0.0	0.2	<0.001	
	Physical contact	-0.5±0.1	-0.2	0.001	
	Self-esteem	-0.3±0.1	-0.1	0.002	

#### BRQ

The *Importance of Interoceptive Awareness* subscale had a mean total score of 17.5±4.6 and did not differ between settings or genders; the *Perceived Connection* subscale had a mean total score of 6.8±3.2 and was higher in inpatients compared to outpatients as well as in men when compared to women; and the *Suppression of Bodily Sensations* item had a mean score of 3.9±1.8 and did not differ between settings or genders (Table 5). *Importance of Interoceptive Awareness* was weakly correlated with lower depression and stress; and *Perceived Connection* with lower affective pain and sensory pain on the PPS, pain disability, depression, and stress. The single item *Suppression of Bodily Sensations* was weakly correlated with longer pain duration, higher disability, and stress and moderately with higher depressive symptoms (Table 6). In linear regression analyses, *Importance of Interoceptive Awareness* (*r*^2^ = 0.21) was independently associated with mindfulness (*p*<0.001), self-acceptance (*p* = 0.036), self-esteem (*p* = 0.006), physical contact (*p* = 0.041; Table 7); *Perceived Connection* (*r*^2^ = 0.16) with setting (*p =* 0.020; higher values in inpatients), gender (*p* = 0.007; higher values in men), self-acceptance (*p*<0.001), vitality (*p*<0.001), and negatively with sensory pain on the PPS (*p* = 0.003; Table 7). *Suppression of Bodily Sensations* (*r*^2^ = 0.16) was independently associated with lower self-esteem (*p* = 0.002) and physical contact (*p*<0.001), and higher depression (*p*<0.001; Table 7).

### Responsiveness

Out of 202 patients, 168 completed the mind-body interventions. Patients who dropped-out of the intervention did not differ from those who completed it regarding gender (*p* = 0.722), age (*p* = 0.352), pain intensity (*p* = 0.802), pain duration (*p* = 0.677), BAQ (*p* = 0.674), and BRS (*p* = 0.45–0.782). After participation in the mind-body program, scores on the BAQ and the *Importance of Interoceptive Awareness* subscale of the BRQ significantly increased, while pain intensity and the *Suppression of Bodily Sensation* decreased; *Perceived Connection* did not change substantially (Table 8).

**Table 8:** Sensitivity to change: Body awareness and body responsiveness (mean±standard deviation) before and after a mind-body group program.

	Week 0 (*n* = 202)	Week 10 (*n* = 168)	*P*
**Intensity of pain**	40.7±26.9	35.0±20.5	<0.001
**Body Awareness Questionnaire**	74.4±16.8	79.5±15.9	<0.001
**Body Responsiveness Questionnaire**			
Importance of Interoceptive Awareness	17.5±4.4	19.6±4.3	<0.001
Perceived Connection	6.4±3.2	6.6±2.9	0.518
Suppression of Bodily Sensations	4.1±1.8	3.7±1.7	0.001

## Discussion

Prior research suggests that attention to bodily sensations is associated with maladaptive attentional and coping styles in chronic pain patients. Other research however suggests that the type of attentional focus, or type of response to body sensations, influences the degree of perceived pain in chronic pain patients. Specifically, mindful, non-judgmental and accepting awareness of the sensory aspects of pain has been shown to reduce the unpleasantness of experimental pain [32–35] and distress from chronic pain [7]. It has e.g. been shown that interventions that increase body awareness in patients with chronic pain may alleviate pain intensity [36], and that pain relief is associated with decreases in catastrophizing, and increases in body awareness [37].

The current research validated two measures of body awareness in German to examine the association between general tendencies of self-reported aspects of body awareness and pain perceptions and disability and sensitivity to a mind-body intervention in a large sample of mainly female inpatient and outpatient adults suffering from chronic pain.

The BAQ showed high internal reliability (alpha = .84) and good validity in line with analogous studies undertaken on the original English version of the BAQ and translations, specifically, a low to moderate positive relation between the BAQ and indicators of valuing body awareness, bodily self-acceptance, and practices associated with mindfulness and no or negative relation to negative moods [38–41].

The Body Responsiveness Questionnaire (BRQ) showed moderate internal reliability and convergent validity. Both subscales of the BRQ were moderately related to greater levels of self-acceptance and mindfulness and less depressive and stress symptoms, suggesting that the BRQ may reflect mindful and accepting responses to bodily sensations.

BAQ and BRQ showed different associations with perceptions of sensory pain and pain-related variables. Higher scores on the BAQ, a measure of self-reported awareness of non-pain-related body sensations, were independently associated with greater depressive symptoms and higher perceived stress, but notwith sensory pain. This is in line with the long-hold view that body awareness is associated with rumination, catastrophizing, and somatization [2], and might thus potentially increase the risk of chronic pain [3]. Higher scores on the *Perceived Connection* scale of the BRQ, in contrast, were weakly associated with lower ratings for sensory pain; and higher scores in *Suppression of Bodily Sensations* (indicating a low body responsiveness) were associated with greater depressive symptoms. Taken together, these findings align with studies of mindfulness and pain perception, which indicate that greater acceptance of and mindful responsiveness to bodily sensations is associated with improved pain outcomes.[references]

Interestingly, the BAQ and *Perceived Connection* subscale of the BRQ were negatively correlated, such that greater awareness of bodily sensations (as assessed by the BAQ) among pain patients was associated with less perceived connection, or perhaps a greater sense of conflict with bodily desires (e.g., to rest, withdraw, or not work). At the same time, however, there was a robust positive correlation between the BAQ and the *Importance* subscale of the BRQ (*r* = .45) and no relation between the BAQ and the *Suppression* item of the BRQ.

Overall, these findings support the complex literature on body awareness and pain. On the one hand, simple attention to and awareness of body sensations among patients with chronic pain without a history of mind-body practice is linked to a sense of mind-body disconnection and increased mental distress. On the other hand, the degree to which individuals value becoming aware of how their body feels and use this formation to regulate behavior–perhaps akin to mindful awareness of body sensations in terms of an open, accepting attitude towards sensations–the greater the reductions in the sensory and affective aspects of pain. Thus, the type of attentional focus to bodily sensations may determine the impact of body awareness on pain perception. Of note, inpatient compared to outpatient participants reported greater levels of perceived connection between mental and physical states. It is not clear why, but perhaps less conflict between mental and physical states allows individuals to seek medical care. It is important to note that most correlations are rather weak and cross-sectional and, therefore, causal relations between variables cannot be implied.

Next we examined the instruments’ responsiveness or sensitivity to change, i.e. whether scores for aspects of body awareness, as assessed by the BAQ and BRQ, increased after a mind-body intervention in outpatient pain patients. Interesting, body awareness and *Importance of Interoceptive Awareness* of the BRQ showed increased levels, but not *Perceived Connection*.

This study has several limitations: first, the sample consisted of participants with a variety of chronic pain conditions. This precludes definite conclusions about body awareness in specific pain conditions. Second, the sample further consisted of over 90% female participants, limiting generalizability. Third, we did not assess how many of the approached patients agreed to participate in our study. Finally, the longitudinal data were uncontrolled, which precludes any causal inferences.

It should also be noted, that the view that body awareness represents a uniform construct has been challenged in the past. Based on phenomenological enquiries and content analyses of available instruments measuring body awareness, a four-dimensional construct has been proposed: i) perceived bodily sensations, ii) attentional quality, iii) attitude to body, and iv) mind-body integration [1,8,42]. While the BAQ and the BRQ mainly cover the dimensions of perceived bodily sensations, attitude to body and mind-body integration, respectively [8], newer instruments such as the *Multidimensional Assessment of Interoceptive Awareness (MAIA)*, which was not yet available at the start of the study, cover all four dimensions [42].

Future research should investigate relations of body awareness and body responsiveness with pain variables in specific pain conditions as well as in more sociodemographically-diverse populations. Further, randomized controlled trials are needed to draw definite conclusions on changes of body awareness by mind-body interventions. Finally, mechanistic studies should investigate whether increased body awareness actually is a mechanism by which mind-body intervention can reduce pain.

In conclusion, body awareness and body responsiveness are associated with pain-related variables in patients with chronic pain. Mind-body interventions may positively influence both, pain and body awareness, hinting at a potential mechanism of action of these interventions.

## Supporting information

S1 Data Set Complete data set. Data on employment and disability were deleted to disidentify patients as per journal request.
(SAV)
